# DARU‐Net: A dual attention residual U‐Net for uterine fibroids segmentation on MRI

**DOI:** 10.1002/acm2.13937

**Published:** 2023-03-29

**Authors:** Jian Zhang, Yang Liu, Liping Chen, Si Ma, Yuqing Zhong, Zhimin He, Chengwei Li, Zhibo Xiao, Yineng Zheng, Fajin Lv

**Affiliations:** ^1^ State Key Laboratory of Ultrasound in Medicine and Engineering College of Biomedical Engineering Chongqing Medical University Chongqing China; ^2^ Chongqing Key Laboratory of Biomedical Engineering Chongqing Medical University Chongqing China; ^3^ Department of Radiology The First Affiliated Hospital of Chongqing Medical University Chongqing China; ^4^ Institute of Medical Data Chongqing Medical University Chongqing China

**Keywords:** attention mechanism, deep learning, segmentation, U‐Net, uterine fibroid

## Abstract

**Purpose:**

Uterine fibroid is the most common benign tumor in female reproductive organs. In order to guide the treatment, it is crucial to detect the location, shape, and size of the tumor. This study proposed a deep learning approach based on attention mechanisms to segment uterine fibroids automatically on preoperative Magnetic Resonance (MR) images.

**Methods:**

The proposed method is based on U‐Net architecture and integrates two attention mechanisms: channel attention of squeeze‐and‐excitation (SE) blocks with residual connections, spatial attention of pyramid pooling module (PPM). We did the ablation study to verify the performance of these two attention mechanisms module and compared DARU‐Net with other deep learning methods. All experiments were performed on a clinical dataset consisting of 150 cases collected from our hospital. Among them, 120 cases were used as the training set, and 30 cases are used as the test set. After preprocessing and data augmentation, we trained the network and tested it on the test dataset. We evaluated segmentation performance through the Dice similarity coefficient (DSC), precision, recall, and Jaccard index (JI).

**Results:**

The average DSC, precision, recall, and JI of DARU‐Net reached 0.8066 ± 0.0956, 0.8233 ± 0.1255, 0.7913 ± 0.1304, and 0.6743 ± 0.1317. Compared with U‐Net and other deep learning methods, DARU‐Net was more accurate and stable.

**Conclusion:**

This work proposed an optimized U‐Net with channel and spatial attention mechanisms to segment uterine fibroids on preoperative MR images. Results showed that DARU‐Net was able to accurately segment uterine fibroids from MR images.

## 1 INTRODUCTION

Uterine fibroid is a very common benign tumor, affecting up to 80% of all women in their reproductive age.^1^ Focused ultrasound surgery is a new and widely used noninvasive method for the treatment of uterine fibroids.^2^ Accurate segmentation of the uterine fibroids is an important part for guiding the therapy preoperatively, and doctors can clearly determine the location, shape, and size of uterine fibroids through magnetic resonance imaging (MRI). Uterine fibroids segmentation usually relies on manual labeling by radiologists, but this repeated mechanical labor is too time‐consuming and laborious, so the segmentation results are easily affected by individual differences. Thus, automatic uterine fibroids segmentation from MR images become an essential issue that is needed to be solved in clinic.

In literature, some methods for uterine fibroids segmentation in MR images have been proposed. In terms of conventional image processing technique, Khotanlou et al. proposed several different methods to segment fibroids on MR images: 1) Using Fuzzy C‐Means method in T1W‐enhanced images and then some morphological operations were applied to refine the initial segmentation.^3^ 2) On the basis of,^3^ applying new Modified Possibilistic Fuzzy C‐Means (MPFCM) algorithm.^4^ 3) A two‐stage method^5^ combing the region‐based level set of Chan‐Vese and the hybrid Bresson methods. Rundo et al.^6^ proposed a method for the segmentation of fibroids on MR images based on an improved direct region detection model. After that, they proposed a novel fully automatic method based on the unsupervised Fuzzy C‐Means clustering and iterative optimal threshold selection algorithms for uterus and fibroid segmentation.^7^ In recent years, due to the wide application of convolutional neural networks (CNNs) in computer vision, many diseases are gradually segmented by CNNs instead of traditional methods. Kurata et al.^8^ used a fine‐tuned U‐Net^9^ to perform uterus segmentation and it achieved a good result. Zhang et al.^10^ and Tang et al.^11^ used the Encoder‐Decoder architecture based on Resnet101,^12^ and they obtained satisfactory uterine fibroids segmentation.

Furthermore, many medical image segmentation networks are based on U‐Net now. U‐Net captures features and reduces spatial size via convolution layers and max pooling players, and recovers object details and spatial size by skip connections, convolution layers, and upsampling operations. It has an excellent performance in the segmentation of some organs and corresponding diseases, such as breast and fibro‐glandular tissue,^13^ prostate,^14^ kidney,^15^ liver, and liver lesions.^16^ U‐Net is simple and effective, but the span of these multi‐stage long skip connections is too large so that it induces a loss of semantic information leading to redundant feature maps. In order to make up for the defect of U‐Net, we optimize U‐Net through deleting invalid information and combining local and global information.

In this paper, we proposed a deep learning model, called dual attention residual U‐Net (DARU‐Net), an optimized U‐Net composed of convolutions with residual connections, channels attention modules, and spatial attention modules, to improve the segmentation of automatic methods in uterine fibroids. We were inspired from the method proposed by Hu et al.,^17^ they use SE blocks to strengthen important features and weaken unimportant ones in channel dimension. In addition, in the spatial dimension, we enhanced the relationship between pixels by residual connections and PPM.^18^

## 2 METHODS

### 2.1 Neural network architecture

**FIGURE 1:**
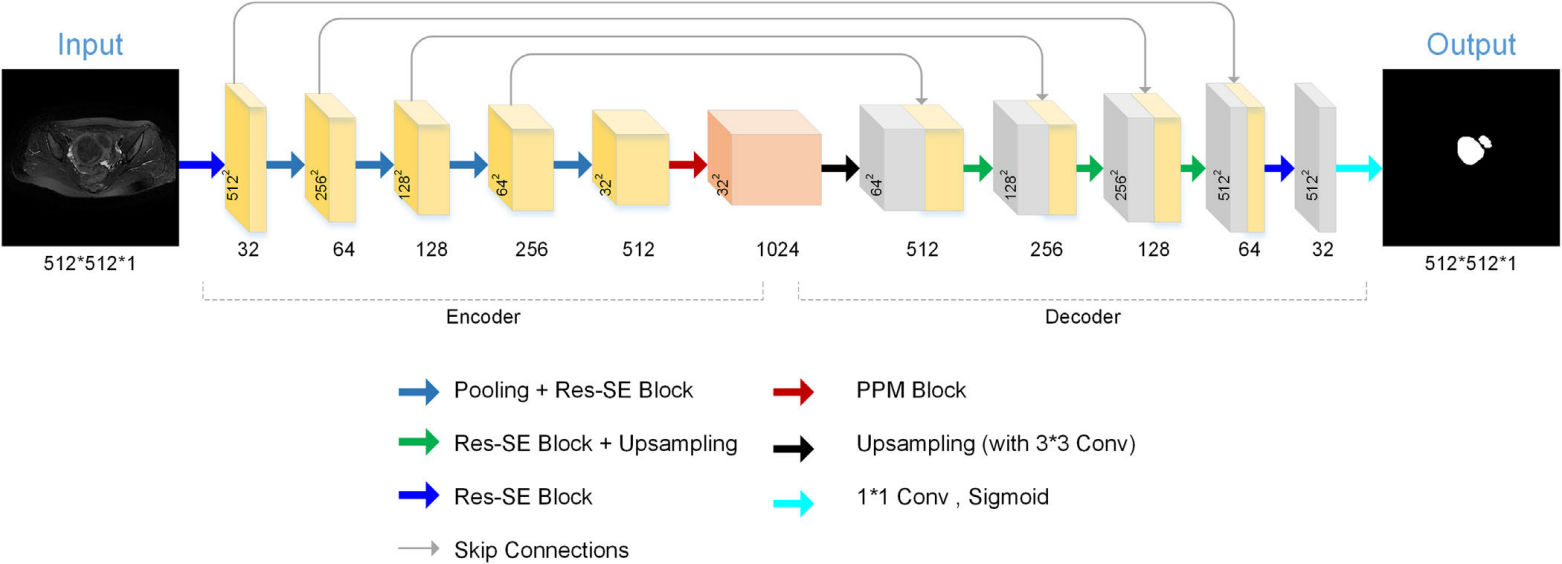
Structure of DARU‐Net.

The model we proposed consists of three parts: the feature encoder module, the feature decoder module, and the PPM. As illustrated in Figure 1, an image is used as the input of the contracting path made up of multiple Res‐SE blocks to extract deep features of image; the size of feature maps reduced step by step via a hybrid pooling layers, which is the sum of average pooling and max pooling. In the deepest part of the network, the feature maps gather spatial information through PPM. Finally, spatial information and edge information of segmentation objects are restored via upsampling layers in the expansive path and skip connections.

### 2.2 Res‐SE block

In CNNs, input images are processed into multiple feature maps after convolution operation. These feature maps all have the same weight, but in fact they are not equally important to segmentation results. So we have to assign different weights to them so that we can suppress the useless parts. SE block^17^ is a concise module to study the correlation between channels, and we introduced it after two 3 × 3 convolutions in the encoder and the decoder to increase the fitting ability of network module. We used global average pooling as the squeeze operation and two fully connected layers as the excitation operation. The dimensionality reduction factor used in the first fully connected layers is 8.

**FIGURE 2:**
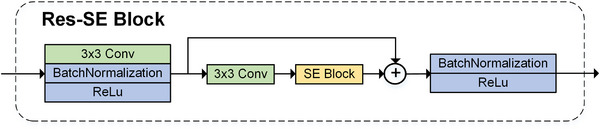
Schematic diagram of Res‐SE block.

At the end, we combined deep and shallow features through a residual connection, which can keep the original information intact, speed up model convergence, and fuse high‐dimension and low‐dimension features together.^19^, ^20^ Figure 2 gives a schematic diagram of Res‐SE block.

### 2.3 Pyramid pooling module

**FIGURE 3:**
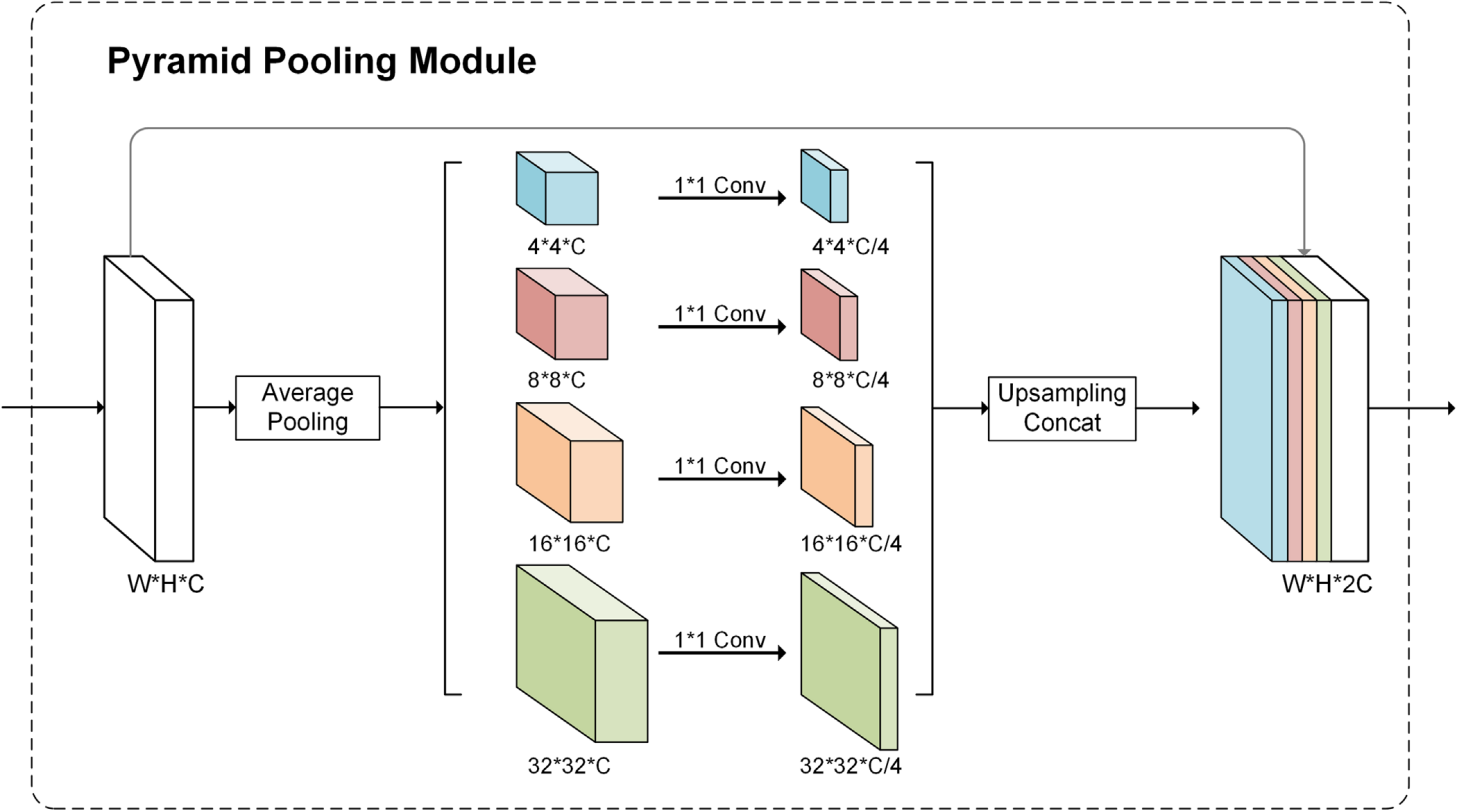
Pyramid Pooling Module structure diagram.

In CNNs, the size of receptive field represents the content of input image information. With the stacking of convolution layers, we used a larger receptive field and more information in each calculation, but we still used the local receptive field. In order to include the global view in the calculation, we used Zhao et al.^18^ that proposed a hierarchical global prior, containing information with different scales and varying among different sub‐regions for reference. They proposed a useful module called PPM, and it mainly consists of four operations: global average pooling of the deepest feature map of the convolutional neural network at four different scales, followed by 1 × 1 convolutions blocks, upsampling layers, and the four feature maps are concatenated on the channel dimension at last. Figure 3 shows the structure of it. The location of uterine fibroids is usually in the center of the image, for adapting our datasets, the pooling kernel is changed from 1,2,3,6 to 4,8,16,32. Different kernels can help the module to capture the spatial information between global pixels and distribute the weight of object to the segmentation results.

## 3 EXPERIMENTS

### 3.1 Experimental settings

This study included patients who underwent MRI examination of the pelvis in the Department of Radiology, the First Affiliated Hospital of Chongqing Medical University from 25 September 2013 to 10 February 2018. We used 150 female patients’ T2 weighted MR images for automatic segmentation. The dataset was randomly divided into a training set of 120 cases and a test set of 30 cases. This study was approved by the ethics review board of our hospital. All cases were obtained from a 3.0T MR unit (GE Signa HDxt 3.0T) with an eight‐channel phased‐array coil, and MRI scan parameters are shown in Table 1.

**TABLE 1:** MRI scan parameters.

Parameter	Value
Slice thickness	6 mm
Slice gap	1.7 mm
Matrix	512*512
Repetition time	4080 ms
Echo time	104.24 ms
Field of view	38 cm*38 cm

All 150 cases were divided into four parts and each part was independently labeled by one radiologist with 5 years of clinic diagnosis experience in the radiology department using ITK‐SNAP (version 3.8.0, http://www.itksnap.org/). After labeling, each radiologist checked the labeled cases by other three radiologists and corrected it. Finally, all the labeled data were checked and corrected by a radiologist with 10 years of clinic diagnosis experience. These labeled areas are regarded as the gold standard of uterine fibroids segmentation. Each case contains approximately 22 axial slices. In order to maintain the proper proportion (7:3) of positive and negative samples, we randomly removed some images without uterine fibroids in the training set. Before training, we used the contrast limited adaptive histogram equalization (CLAHE) to enhance the local contrast, and data augmentation (vertical and horizontal flip, ±15° rotation, ±10% x‐axis shift, ±10% y‐axis shift) was applied.

We start training the model with a minibatch size of 5 on an NVIDIA GeForce RTX 2080 Ti GPU with 11GB memory. We use the Adam^21^ optimizer and set the learning rate to 2e‐4. Dice loss function was used as the cost function.^22^ The programming language we used is Python, and the convolutional neural networks are implemented on the platform of Keras (version 2.3.1).

### 3.2 Experimental design

To verify the effectiveness of Res‐SE blocks and PPM, we did the ablation study in our experiments. Initially, we used U‐Net as the baseline. Then we added Res‐SE blocks and PPM respectively. After that, these two modules were combined on the baseline. For more detailed study, we divided the Res‐SE block into two parts: only residual connections added and only SE blocks added. At last, we compared DARU‐Net with other advanced deep learning methods, including Attention‐UNet,^23^ LEDNet,^24^ HRNet,^25^ and MultiResUNet.^26^ We performed 5‐fold cross validation during training, and used the average value as the evaluation of performance. We verified the performance of each model on the same dataset.

### 3.3 Evaluation metrics

In this study, manual segmentation label were served as the ground truth for performance comparisons. By comparing the segmentation results with the ground truth, three basic indicators in statistics were calculated: TP (True Positive), TN (True Negative), FP (False Positive), and FN (False Negative). Based on these indicators, we could get four evaluation metrics: DSC, precision, recall, and Jaccard index. The four metrics mentioned above are defined as follows:

(1) $$DSC=\frac{2*TP}{2*TP+FP+FN}$$

(2) $$Precision=\frac{TP}{TP+FP}$$

(3) $$Recall=\frac{TP}{TP+FN}$$

(4) $$Jaccard\;index=\frac{TP}{TP+FP+FN}$$

## 4 RESULTS

In this section, we have shown the segmentation results of each model mentioned in Experimental Design. The performance measure and the number of trainable parameters of ablation study are shown in Table 2. U‐Net achieved a mean DSC of 0.7563 ± 0.1407. In the experiment of adding residual connections and SE blocks separately, both modules improved recall and JI, but the model with SE blocks had a decrease in precision. Res‐SE blocks improved the mean DSC by 2.56%, as well as increased stability by reducing the standard deviation from 0.1407 to 0.1277. PPM also had improvement in four metrics. The average DSC, precision, recall, JI of DARU‐Net were measured as 0.8066, 0.8233, 0.7913, 0.6743. By combining Res‐SE blocks and PPM, we can not only achieve higher segmentation accuracy, but also obtain more stable segmentation results which can be seen in the gradually decreased standard deviation (std). The increase in the number of trainable parameters is mainly due to PPM.

**TABLE 2:** Comparison of results obtained by different models in the ablation study

	DSC(%)	Precision(%)	Recall(%)	JI(%)	Params(M)
U‐Net	75.63 ± 14.07	81.68 ± 13.98	73.42 ± 18.38	62.63 ± 16.10	**8.63**
U‐Net + Res^a^	76.61 ± 13.80	81.79 ± 12.94	75.83 ± 20.07	64.18 ± 17.09	8.63
U‐Net + SE^b^	77.51 ± 14.57	79.58 ± 13.83	78.49 ± 18.24	65.27 ± 16.55	8.74
U‐Net + Res‐SE	78.19 ± 12.77	**82.81** ± **10.81**	76.62 ± 17.11	65.76 ± 15.11	8.74
U‐Net + PPM	77.94 ± 10.98	82.72 ± 11.94	76.13 ± 16.19	65.22 ± 14.60	10.07
DARU‐Net	**80.66** ± **9.56**	82.33 ± 12.55	**79.13** ± **13.04**	**67.43** ± **13.17**	10.18

Results were represented as the mean value ± standard deviation; The best results are highlighted in bold.

^a^ Only residual connections added.

^b^ Only SE blocks added.

The experimental results of each other deep learning methods are summarized in Table 3, and DARU‐Net outperforms the other segmentation methods. In addition to precision, DARU‐Net has better performance than other deep learning methods in the rest four metrics. LEDNet has an obvious improvement in precision but weak performance in recall. HRNet and MultiResUNet cannot obtain a higher DSC scores than U‐Net from their dense connections. Only the Attention U‐Net increases the DSC through its attention gates.^22^ In terms of the number of trainable parameters, DARU‐Net improves the accuracy and stability by adding few parameters.

**TABLE 3:** Comparison of results obtained by different deep learning methods

	DSC(%)	Precision(%)	Recall(%)	JI(%)	Params(M)
U‐Net	75.63 ± 14.07	81.68 ± 13.98	73.42 ± 18.38	62.63 ± 16.10	8.63
Attention U‐Net	77.23 ± 13.55	82.61 ± 14.60	75.84 ± 18.84	64.99 ± 17.20	8.97
LEDNet	73.09 ± 12.65	**87.12** ± **10.80**	65.16 ± 16.36	59.20 ± 15.18	**4.94**
HRNet	74.53 ± 17.36	77.83 ± 18.90	74.52 ± 20.43	61.81 ± 17.61	9.50
MultiResUNet	75.43 ± 14.90	82.93 ± 13.92	72.78 ± 20.18	62.91 ± 17.99	6.88
DARU‐Net	**80.66** ± **9.56**	82.33 ± 12.55	**79.13** ± **13.04**	**67.43** ± **13.17**	10.18

Results were represented as the mean value ± standard deviation; The best results are highlighted in bold.

**FIGURE 4:**
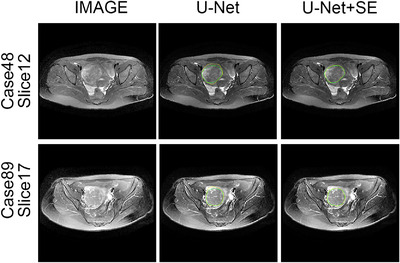
Segmentation results by using U‐Net and U‐Net with the SE blocks. The left is raw MR image. The middle and right are the segmentation results of two methods (Green Contour Circle: fibroids ground truth, Yellow Contour Circle: segmented fibroids region).

**FIGURE 5:**
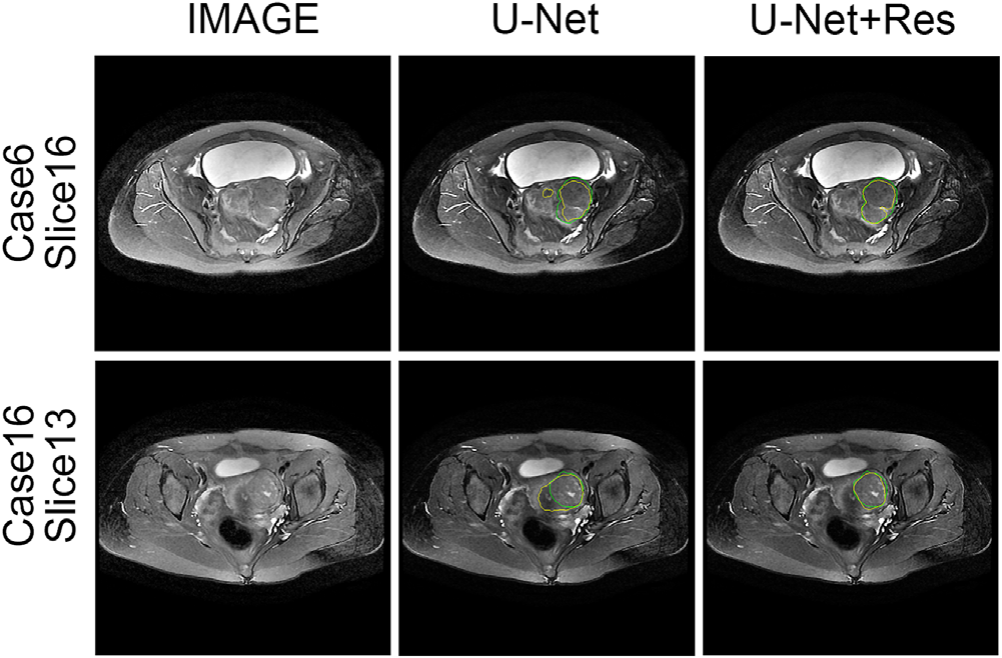
Segmentation results by using U‐Net and U‐Net with residual connections. The left is raw MR image. The middle and right are the segmentation results of two methods (Green Contour Circle: fibroids ground truth, Yellow Contour Circle: segmented fibroids region).

**FIGURE 6:**
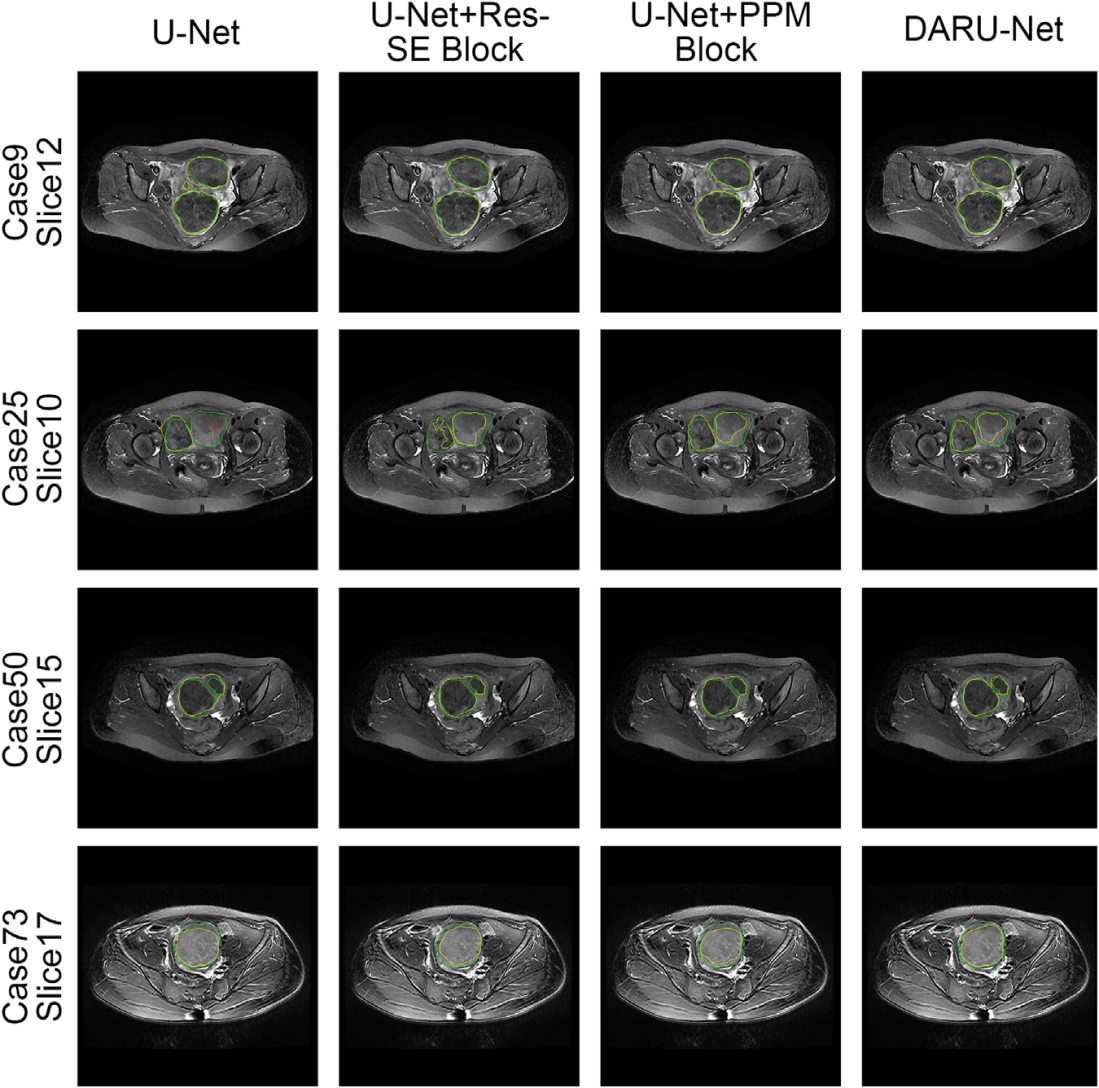
Comparison of segmentation of U‐Net, U‐Net with Res‐SE block, U‐Net with PPM and DARU‐Net (Green Contour Circle: fibroids ground truth, Yellow Contour Circle: segmented fibroids region).

**FIGURE 7:**
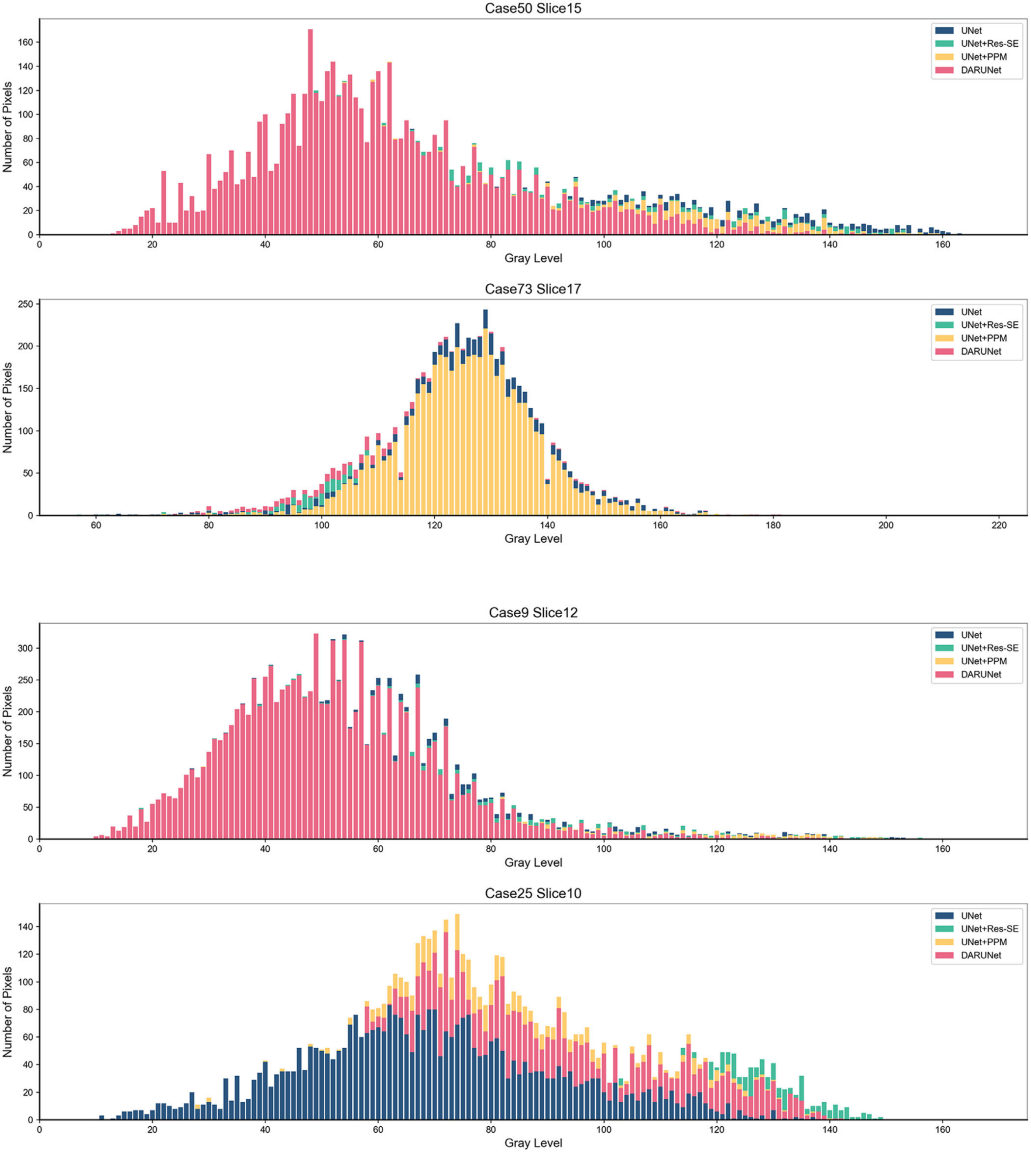
Histogram of the signal intensity of segmented area obtained through different methods.

The quantitative experimental results suggest that DARU‐Net has better learning ability and performance on segmentation of uterine fibroids than the original U‐Net and other deep learning segmentation methods. Some visual segmentation results are shown to demonstrate the improved segmentation capabilities of DARU‐Net. In Figure 4, U‐Net with SE blocks shows better recognition of low signal intensity regions. It can be seen from Figure 5 that U‐Net with residual connections is able to find the correct boundary of uterine fibroids. Figure 6 illustrates PPM plays the same important role as Res‐SE block in improving segmentation quality. Figure 7 shows the gray histogram of segmentation results in some actual cases, and the corresponding image is shown in Figure 6. It can be seen from case 50 and case 73 that with the addition of attention mechanism, the segmentation results become more accurate. The number of hyperintensity pixels in the segmentation result decreases while the number of hypointensity pixels increases.

**FIGURE 8:**
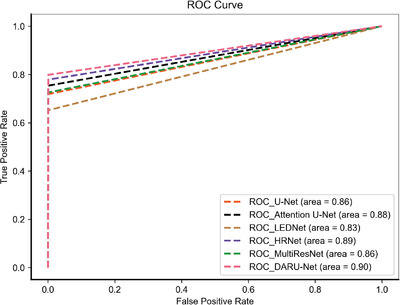
ROC Curve of different deep learning methods on the test set.

Some automatic segmentation results are shown in Figure 6. It can be seen that the original U‐Net has a basic ability to capture uterine fibroids, but it cannot perform well in such as the edge of the fibroids and area where signal strength changes. With Res‐SE block and PPM added, the model performs better in detail segmentation and some difficult tasks. The original U‐Net sometimes misjudges normal tissue as fibroids like case 9, and after optimized, DARU‐Net can avoid such situations through filtering out some hyperintensity pixels. In case 25, the fibroid shows unbalanced signal intensity area, and U‐Net cannot identify it well. There is a yellow point in the right area (marked by a red arrow), which shows that U‐Net has the ability to identify the pixels in this area, but it is very little. As it is shown in case 50, with the addition of Res‐SE block and PPM, the results are closer to the labelled outline drawn by the radiologists. In case 73 which contains fibroids with high signal, DARU‐Net also achieves the best segmentation results. Figure 8 shows the ROC curve of these models, DARU‐Net has the largest area under the curve.

**FIGURE 9:**
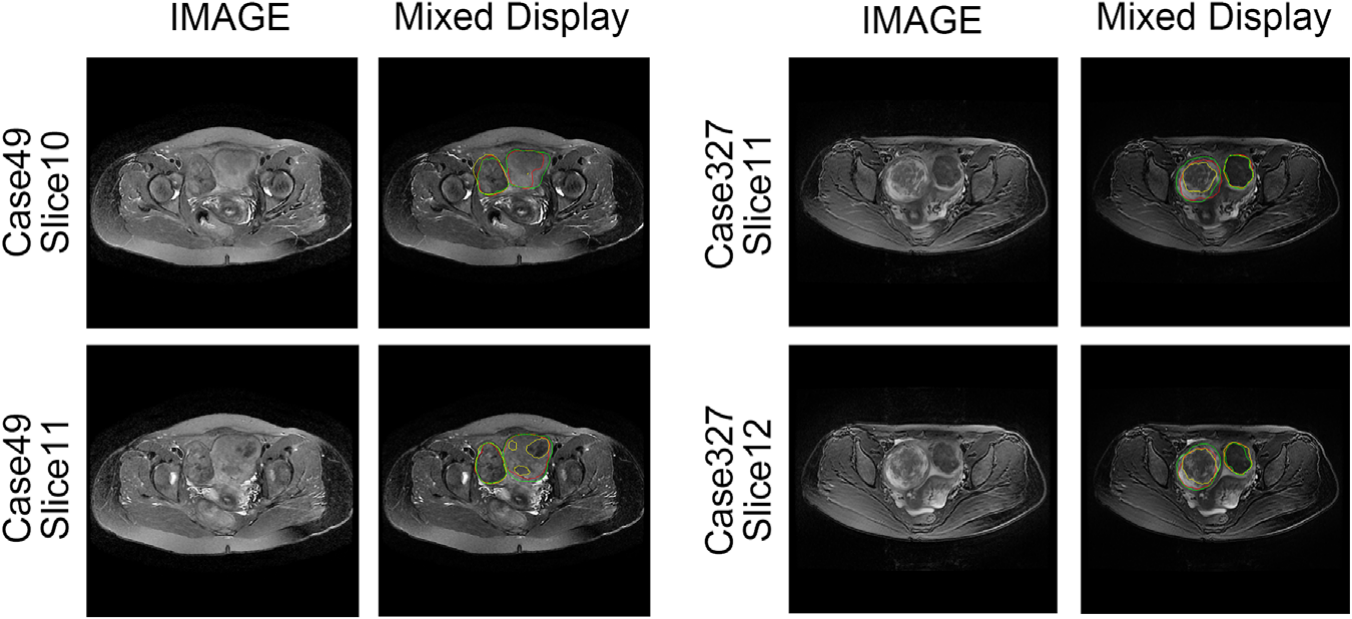
Comparison of segmentation of U‐Net and DARU‐Net in cases with different SNRs (Green Contour Circle: fibroids ground truth, Red Contour Circle: segmented fibroids region in DARU‐Net, Yellow Contour Circle: segmented fibroids region in U‐Net).

**FIGURE 10:**
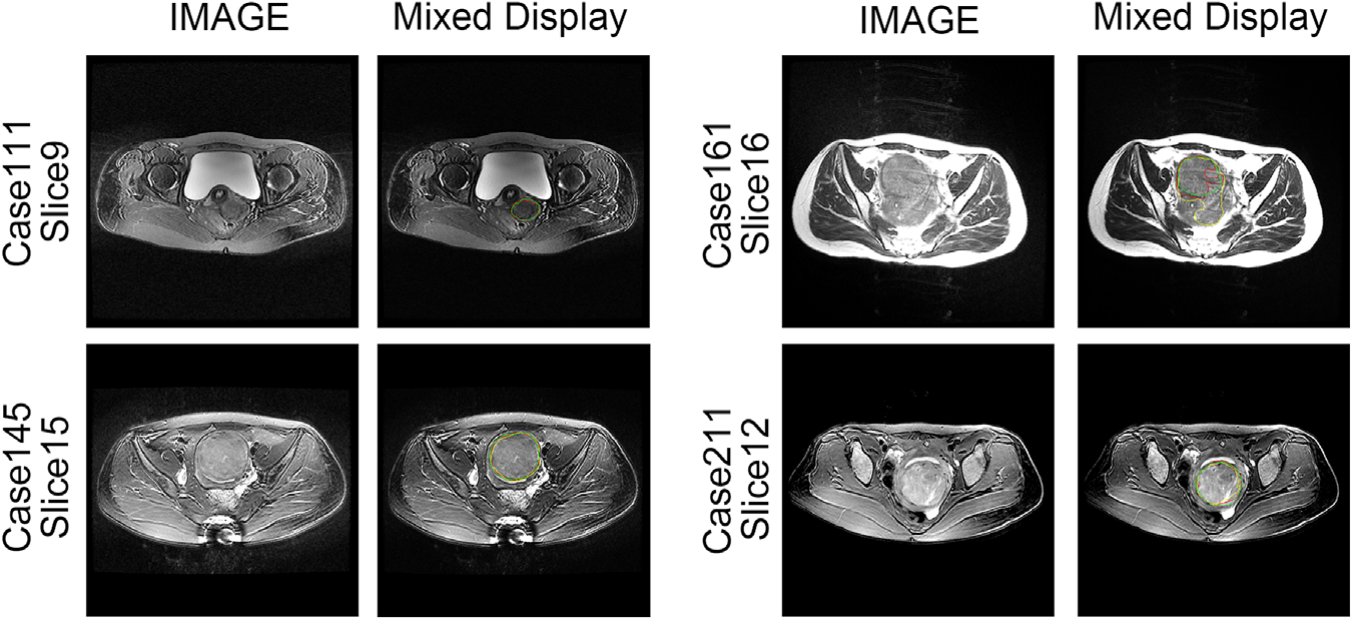
Comparison of segmentation of U‐Net and DARU‐Net in cases with motion artifacts (Green Contour Circle: fibroids ground truth, Red Contour Circle: segmented fibroids region in DARU‐Net, Yellow Contour Circle: segmented fibroids region in U‐Net).

Some cases with the region of different signal‐to‐noise ratio (SNR) are shown in Figure 9. It's clearly that DARU‐Net has better recognition ability for tumor regions with different SNRs. Figure 10 shows the recognition capability of the model in cases with motion artifacts. DARU‐Net performs better than U‐Net in the case of tumor artifacts and low signal intensity regions. The influence of artifacts in non‐interest region is almost the same.

**FIGURE 11:**
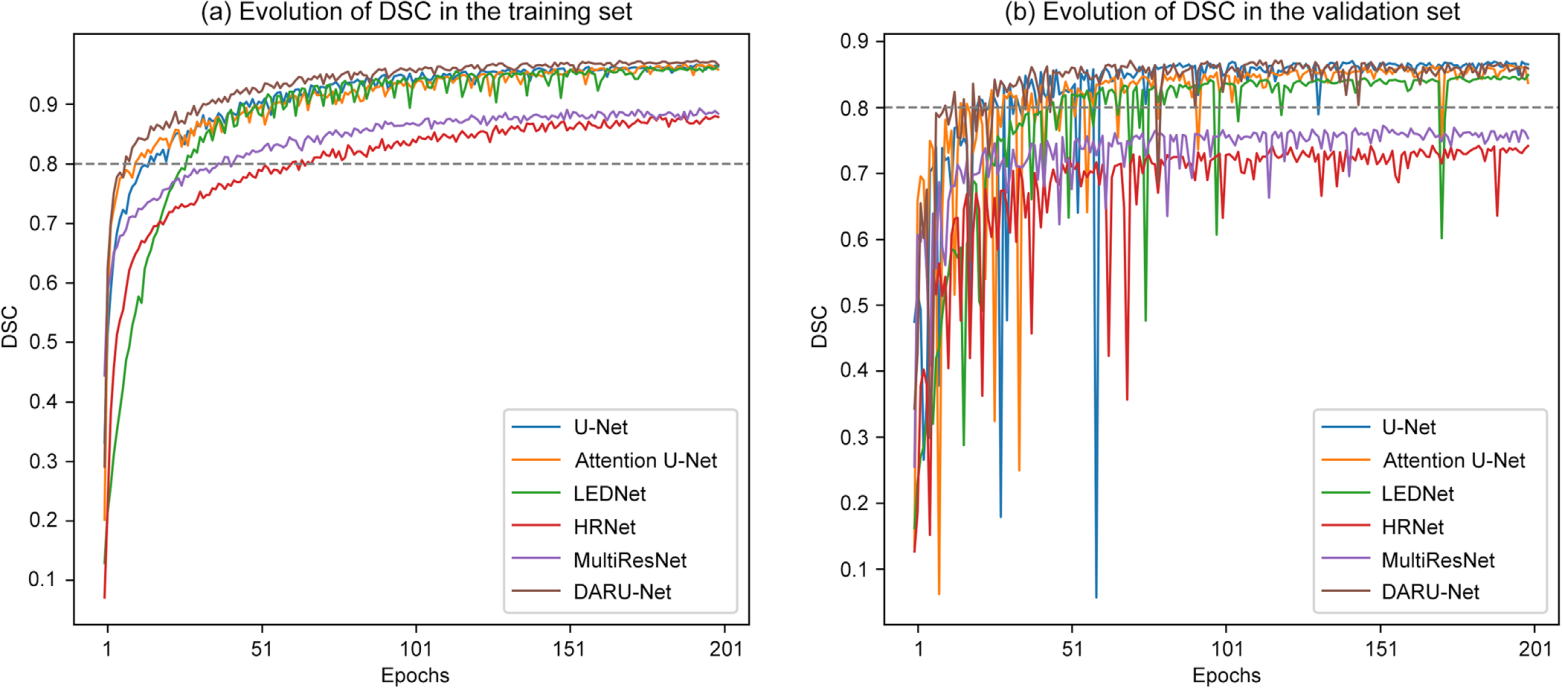
DSC curves in training set (a) and validation set (b) during training different models.

Figure 11 shows the evolution of DSC for the training and validation set, respectively. DARU‐Net has faster convergence rate compared to other deep learning models, reaching a DSC of 0.8 in 7 epochs in the training data and 10 epochs in the validation data.

**FIGURE 12:**
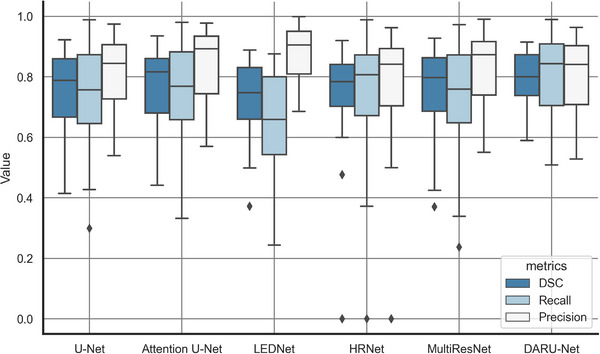
Boxplot of DSC, recall and precision for all test cases in different deep learning models.

As shown in Figure 12, DARU‐Net has the second highest median DSC and the smallest data fluctuation. Except DARU‐Net and Attention U‐Net, all other models have outliers. In DSC, the median and upper quartile of DARU‐Net and Attention U‐Net are very close (0.8002(0.7387,0.8734) and 0.8165(0.6808,0.8608)), but DARU‐Net has higher lower quartile. LEDNet has high precision, but low recall. It adopts ResNet as backbone network and utilizes channel split and shuffle in each residual block. These operations enlarge network capacity, so it has higher precision. But it didn't consider the connections between encoder and decoder, which leads many true positive pixels to be lost in the long convolution process. On the whole, DARU‐Net has the best stability in the prediction results among them.

## 5 DISCUSSION

This paper mainly studied the MRI automatic segmentation of uterine fibroids based on deep learning model. In previous literature, researchers usually used unsupervised learning method, such as Fuzzy C‐Means and its optimized algorithm,^3^, ^4^, ^7^ and they achieved the segmentation of uterine fibroids on MR images through clustering algorithm and image post‐processing methods. These studies demonstrated that uterine fibroids can be identified and segmented on T2 weighted MR images. The proposed model, DARU‐Net, is based on U‐Net, and it improves the segmentation results after introducing two attention mechanisms. It proves that introduction of spatial attention and channel attention mechanism is of great help in identifying uterine fibroids.

In most images, the signal intensity of uterine fibroids is usually lower than surrounding tissue, and SE blocks help assign weights to correct low signal intensity feature maps, so that high intensity signal regions receive less attention and segmentation results are improved.

The residual connections can locate the target accurately according to context information from residual connections which links deep and shallow features maps, so the edge of uterine fibroids can be well detected and drawn. Distinct improvement of recall shows that these two methods both can improve the segmentation results effectively via finding more true positive points from the MR images. The SE blocks and residual connections are united to avoid a drop in precision and keep the improvement in recall. In contrast, PPM obtains context information with a larger receptive field and fuses the features at different scales rather than linking up the same‐scale feature maps, and forms the final feature representation through upsampling and concatenation layers, which carries both local and global context information. Res‐SE block and PPM have a better ability to identify areas with large gray changes, so DARU‐Net can identify most areas of the fibroid.

In real cases, there are some interferences in MR images. For example, the signal intensity of intestinal tracts or other surrounding tissues is similar to uterine fibroids, and different signal strengths in local areas lead to different SNRs. Motion artifacts also have an impact on image quality. To solve these problems, we combined the three methods mentioned above to form DARU‐Net. This optimized U‐Net collected semantic information from spatial and channel domain.

We compared our method with five deep learning method, including U‐Net, Attention U‐Net, LEDNet, HRNet, and MultiResUNet. U‐Net is the baseline model. Attention U‐Net shows an improvement on DSC scores in Table 3, which proves the effectiveness of channels attention mechanisms. As for the unimproved standard deviation of it, we think that is because the span of Attention Gate^23^ connecting the encoder and decoder is too large so that there are abrupt changes in the data. So the robustness of Attention U‐Net has not been improved. LEDNet uses channel split and shuffle in each residual block.^24^ This structure expands the network capacity in a sense without increasing computational cost. However, LEDNet focuses on precision and real‐time performance, but ignores recall. In medicine, false negatives are more unacceptable than false positives. So even though precision of LEDNet is high, the recall of it is too low to make it trustworthy. HRNet does not improve in performance with increasing parameters, and it has many outliers in Figure 12. Because it focus on high‐resolution information, while the medical image is simple in structure, and the deep features are more important. MultiResUNet uses smaller 3 × 3 convolutional kernels to replace 5 × 5 and 7 × 7 convolutional kernels,^26^ and it does not gain performance gains from its multiple residual connections.

There are some limitations in this study. First, these data come from a single‐center, which means all images used in training and testing come from the same region and the same hospital. We need to use multicenter cases to improve the robustness and generalizability of DARU‐Net. Second, we only used T2 weighted MR images for research. We will consider using other multimodal images in the future.

## 6 CONCLUSIONS

In this study, we proposed the DARU‐Net for uterine fibroids segmentation on MR images. DARU‐Net is based on U‐Net and improved with two attention mechanisms: SE blocks with residual connections in channel domain and PPM in spatial domain. Experimental results on a clinical dataset of 150 cases shows that DARU‐Net has better segmentation than the baseline U‐Net and other advanced deep learning methods. DARU‐Net gains average DSC of 0.8066 ± 0.0956, which proves that DARU‐Net has sufficient accuracy and stability, and can be used for automatic segmentation of uterine fibroids in clinic.

## AUTHOR CONTRIBUTIONS

Conceptualization: Fajin Lv, Yineng Zheng; Methodology: Jian Zhang, Yang Liu, Zhibo Xiao; Data curation: Liping Chen, Si Ma, Yuqing Zhong, Zhimin He, Chengwei Li; Investigation: Liping Chen, Si Ma, Yuqing Zhong, Zhimin He, Chengwei Li; Validation: Liping Chen, Si Ma, Yuqing Zhong, Zhimin He, Chengwei Li; Software: Jian Zhang; Formal analysis: Jian Zhang; Visualization: Jian Zhang; Writing ‐ original draft: Jian Zhang; Writing ‐ review and editing: Yang Liu, Zhibo Xiao.

## CONFLICT OF INTEREST STATEMENT

The authors have no relevant conflicts of interest to disclose.
